# Investigation of Quiet Quitting and Professional Quality of Life Among Nursing Staff at a General Hospital: A Single-Site Cross-Sectional Study

**DOI:** 10.3390/healthcare14183004

**Published:** 2026-09-14

**Authors:** Michael Rovithis, Nikolaos Rikos, Kalliopi Zachou, George Kritsotakis, Manolis Linardakis, Lena Borboudaki, Areti Stavropoulou

**Affiliations:** 1 Department of Business Administration & Tourism, School of Management and Economics Sciences, Hellenic Mediterranean University, 71410 Heraklion, Greece; gkrits@hmu.gr; 2 Department of Nursing, School of Health Sciences, Hellenic Mediterranean University, 71410 Heraklion, Greece; rikosn@hmu.gr; 3 Venizeleio & Pananio General Hospital, 71409 Heraklion, Greece; kalizahou@yahoo.gr; 4 Department of Social Medicine, School of Medicine, University of Crete, 70013 Heraklion, Greece; linman@med.uoc.gr (M.L.); lenaborboudaki@uoc.gr (L.B.); 5 Department of Nursing, Faculty of Health Science, West Attica University, 12243 Athens, Greece; astavropoulou@uniwa.gr

**Keywords:** quiet quitting, professional quality of life, burnout, secondary traumatic stress, compassion fatigue, compassion satisfaction, managerial support, nurse

## Abstract

**Background/Objectives:** Quiet quitting and reduced professional quality of life pose risks to organizations, as employee performance may be reduced. This study assessed quiet quitting and professional quality of life among nurses at a public general hospital in Greece. **Methods:** A single-site cross-sectional study recruited 119 nursing staff from a public general hospital in Greece between January and March 2025. Statistical analysis was performed using SPSS version 25.0. Demographic and work characteristics were analyzed using absolute and relative frequency distributions. Comparisons among subscale scores were made using the Kruskal–Wallis test, and associations with participant characteristics were examined using Pearson’s correlation coefficient. Multiple linear regression analyzed the relationships between the three ProQOL-30 subscales, the Quiet Quitting Scale, and nursing staff characteristics, with *p* < 0.05. **Results:** Of the 119 nursing staff, 84.9% were women, and 49.6% were 50 or older. Quiet quitting was moderate to low, with lack of motivation significantly higher than lack of initiative and detachment-indifference (*p* < 0.001). Compassion satisfaction was higher than burnout and secondary traumatic stress (*p* < 0.001). Lack of motivation was higher among nurses with children, more years of experience, or managerial responsibilities (*p* < 0.05). Compassion satisfaction was associated with lower total quiet quitting (unstandardized β = −6.23, *p* < 0.001), while burnout was associated with higher total quiet quitting (β = 4.54, *p* < 0.001). Neither compassion satisfaction nor burnout was related to staff characteristics. Secondary traumatic stress was associated with female gender (β = 4.92, *p* = 0.004) and higher total quiet quitting (β = 3.40, *p* = 0.001). **Conclusions:** Reducing workload, improving organizational support, and enhancing working conditions may represent promising strategies for supporting nurses’ professional quality of life and reducing occupational disengagement.

## 1. Introduction

Quiet quitting mainly refers to employees’ tendency to limit themselves to performing their core duties, as defined by their job description, rejecting the mentality of working overtime and being continuously available for additional work, and adopting the motto “I work to live, not live to work” [1]. Employees aim to work only as long as necessary to avoid being dismissed [1]. Quiet quitting is linked to employees’ need for work–life balance and the protection of their mental health. Workers in the healthcare sector are no exception, and the phenomenon has assumed alarming proportions, as recorded in the international literature [2,3,4].

Quiet quitting should be conceptually distinguished from turnover intention and job disengagement. It involves weakened social connections at work, reduced job engagement, a generalized reluctance toward work, and a desire for clear boundaries between work and personal life, without being equivalent to an explicit intention to leave the organization. Despite similarities between the two concepts, quiet quitting is primarily characterized by psychological distancing at work, and for some nurses it functions as an intermediate coping mechanism for those who wish to leave but are unable to do so [5].

In the modern work environment, quiet quitting has become increasingly common, with 62% of employees worldwide exhibiting this behavior, according to a recent poll [6]. In the health sector, particularly in the nursing profession, demanding and often exhausting working conditions, including high occupational stress and the emotional demands of caring for patients, have attracted particular interest from researchers and administrators and have often been associated with burnout and intention to leave [2,7,8]. These conditions worsened during the COVID-19 pandemic, with nurses facing increased workloads, intense psychological pressure, and a high risk of burnout [9,10]. The phenomenon places organizations at risk, as employees who choose quiet quitting as a work behavior significantly reduce their performance, frequently use sick leave, arrive late to work or stay late without offering new suggestions or ideas, and increase pressure on other employees who take on additional work, resulting in reduced organizational productivity and possible burnout among staff who take on additional work [10,11].

The Job Demands–Resources (JD-R) model [12] provides an appropriate interpretive framework for understanding how working conditions relate to employee disengagement, including quiet quitting, particularly among nursing staff. Under the JD-R model, high demands and insufficient resources have been theorized to increase stress, reduce satisfaction, and are associated with detachment, whereas adequate support and resources maintain job satisfaction even in difficult work situations. High occupational stress, understaffing, shift work, and the emotional strain of patient care have been associated with increased risk of burnout. Burnout have been associated with higher quiet quitting rates among nurses, and work satisfaction has been proposed to mediate the relationship between burnout and quiet quitting, which may function as a coping behavior [13,14]. The JD-R model may serve as a sequential mediating pathway, moderated by individual and organizational factors, including gender, shift work, staffing levels, quality of work life, and organizational support [13,14].

For nurses, the theoretical framework incorporates patient care as an organizational-level outcome. Reduced nurse engagement has been associated with a diminished capacity to meet patient needs during shifts, which may in turn be linked to prolonged hospitalizations, increased risks to patient safety, and higher healthcare costs [3,14,15].

Although quiet quitting has garnered increasing global attention, research specifically examining this phenomenon among nurses remains limited. A cross-sectional study investigated the potential mediating role of turnover intention in the relationship between job burnout and quiet quitting among nurses, surveying 317 nurses from a training and research hospital in Turkey. Notably, 62.5% of nurses identified as quiet quitters [16]. In a similar study involving 205 nurses at a university hospital in Turkey, the mean score on the Quiet Quitting Scale was 2.48 ± 0.65, indicating a significant prevalence of quiet quitting among nurses [17]. A comprehensive systematic literature review and meta-analysis conducted in 2026 included fourteen cross-sectional studies involving 8279 healthcare workers. The reported prevalence of quiet quitting ranged from 46.1% to 74.4%. The studies were conducted in Greece, Turkey, Italy, Egypt, Serbia, and South Korea [18].

In Greece, the economic crisis led to a significant reduction in public health spending and to austerity policies, which weakened the health system and burdened health structures, limiting both access to and the quality of services. Resource and personnel cuts led to hospital understaffing and heavier workloads, particularly for nurses, who were called upon to work under increased pressure amid shortages [19]. A study of nursing staff in Greek hospitals found that seven out of ten nurses described their work stance as quiet quitting, with the highest rates among shift workers and those in understaffed departments [3].

Quiet quitting among nursing staff is directly linked to their professional quality of life. This term “professional quality of life” encompasses not only the mental and physical well-being of healthcare workers but also job satisfaction, work–life balance, workplace safety, and recognition of their contributions. The literature indicates that prolonged exposure to adverse conditions and limited opportunities for rest increase the risk of burnout and secondary traumatic stress, particularly among professionals who directly interact with patients facing severe health challenges [5,20]. Nurses who remain in their positions while reducing their commitment and performance are susceptible to burnout and lower job satisfaction [21]. Burned-out nursing staff are more likely to engage in quiet quitting, with job satisfaction mediating the relationship between burnout and quiet quitting [22]. A study examining the effect of professional quality of life on quiet quitting among health care professionals in public health organizations in central Serbia found that higher professional quality of life may be associated with higher quiet quitting, especially among women [23]. In a relevant study in Ethiopia, healthcare staff with a higher professional quality of life tend to show greater job satisfaction, commitment, and productivity, along with lower absenteeism and staff turnover, which may be associated with improved organizational efficiency and success [24].

Nurses in Greece face a significant risk of burnout and disengagement due to ongoing staff shortages, austerity-related cuts to healthcare funding, demanding work schedules, and the psychological effects of the COVID-19 pandemic. Understanding the phenomenon within the organizational, economic, and cultural contexts of the Greek healthcare system may help inform the development of targeted, evidence-based policies aimed at addressing burnout, supporting job satisfaction and reducing turnover intentions.

In this context, the following six hypotheses were formulated, organized around one primary and three secondary aims described below:

**H1.**  *Nurses will report measurable levels of quiet quitting and professional quality of life.*

**H2.**  *Higher levels of quiet quitting will be associated with poorer professional quality of life.*

**H3.**  *Work-related characteristics will be associated with levels of quiet quitting among nurses.*

**H4.**  *Work-related characteristics will be associated with professional quality of life among nurses.*

**H5.**  *Demographic characteristics will be associated with levels of quiet quitting among nurses.*

**H6.**  *Demographic characteristics will be associated with professional quality of life among nurses.*

The primary aim of the present study was to assess the levels of quiet quitting and professional quality of life among nurses working in a public general hospital in Greece (H1). The secondary aims were to (a) examine the associations between quiet quitting and professional quality of life (H2); (b) assess whether work-related characteristics, such as workload, employment status, work experience, and working conditions, are associated with quiet quitting and professional quality of life (H3; H4); and (c) determine whether demographic characteristics, including age, gender, marital status, educational level, and other relevant sociodemographic factors, are associated with levels of quiet quitting and professional quality of life (H5, H6).

## 2. Materials and Methods

### 2.1. Study Design

This single-site cross-sectional study aimed to investigate quiet quitting and professional quality of life among nurses at a public general hospital in Greece.

### 2.2. Study Population-Survey Instrument

The study population comprised nurses selected through convenience sampling. The inclusion criterion was at least one year of experience. Nurses on extended leave during the data collection period and those in administrative positions (such as exclusively administrative support) were excluded. This hospital was selected because it is one of the largest public general hospitals in the Region of Crete. It functions as a referral center for the broader Heraklion area and beyond, with a capacity of 450 beds. Regarding staffing, the hospital currently has 1048 employees across all services, the lowest number in six years, due to ongoing staff reductions since 2020. By the end of 2025, the nursing service employed 484 nursing staff members, comprising both permanent and contract-based positions. In contrast, the hospital’s official organizational chart enumerates 597 positions. This disparity signifies a substantial staffing shortfall exceeding 100 positions. Furthermore, it was selected over other hospitals on the island due to its systematic documentation of data. Data were collected from January to March 2025. A total of 119 nursing staff members participated in the study. Data were collected face-to-face by the researcher, who employed a systematic approach by visiting hospital clinics and distributing questionnaires to nurses who were on duty on the designated day and time. This process was repeated on the odd days of the month.

We used a structured questionnaire that incorporated two validated standardized research tools for the Greek population, along with demographic questions. The Quiet Quitting Scale consists of nine questions/sentences that estimate the average of three components by estimating the average: detachment (sentences 1, 2, 3 & 4), lack of initiative (5, 6 & 7), and lack of motivation (8 & 9). The total score across all questions is also calculated. Responses are rated on a 5-point Likert scale (1: strongly agree to 5: strongly disagree), with propositions 7–9 reversed. A higher average across all questions indicates a higher level of tacit withdrawal. An overall score of 2.06 or higher indicates tacit withdrawal [25].

The 30-item Professional Quality of Life Scale (ProQOL, 5th edition, 2009) [26,27] was also used to assess three components: compassion satisfaction (questions 3, 6, 12, 16, 18, 20, 22, 24, 27, 30), burnout (questions 1, 4, 8, 10, 15, 17, 19, 21, 26, 29), and secondary traumatic stress (questions 5, 7, 9, 11, 13, 14, 23, 25, 28) [24]. When calculating scores, the 5-point Likert scale (1: never to 5: very often) is reversed for questions 1, 4, 15, 17, and 29. We defined cut-offs as ≤21 (low), 22–41 (medium), and 42+ (high) for satisfaction, burnout, and stress, respectively. In general, higher scores indicate greater compassion but also greater burnout and stress. For both scales, the present study observed very good response reliability (see Table 4).

### 2.3. Ethics

Prior to study initiation, ethical approval was obtained from the Scientific Board of the 7th Health Region of Crete (Ref. No. 48992, approval date: 25 November 2024). All procedures followed the ethical standards of the institutional research committee and the Declaration of Helsinki (revised 2013) [28]. Each participant received comprehensive written and oral information about the study’s objectives, methods, potential risks, and anticipated benefits. The researchers obtained written informed consent from all participants before data collection began. The study emphasized voluntary participation and informed participants of their right to withdraw at any time without penalty.

### 2.4. Statistical Analysis

The analysis of the survey data was conducted using SPSS (IBM Corp., 2019, IBM SPSS Statistics for Windows, v.25.0, Armonk, NY, USA). Frequency distributions of the nursing staff’s demographic and work characteristics were calculated. To assess the consistency of responses on the Quiet Quitting Scale and the Professional Quality of Life Scale (ProQOL-V), Cronbach’s reliability coefficients were also calculated. Scale and subscale scores were assessed for normality using the Blom method (Q-Q plot) and by estimating asymmetry coefficients. Correlations with participant characteristics were assessed using the Pearson correlation coefficient with 95% confidence intervals (95%CIs). Multiple linear regression was also applied to examine relationships between the scores of the three subscales of the Professional Quality of Life (ProQoL-V) scale and the characteristics of the 119 participating nursing staff, as well as the Quiet Quitting Scale. The acceptable significance level was set at 0.05.

## 3. Results

### 3.1. Demographic and Work-Related Characteristics of Nurses

Of the 119 nurse participants, 84.9% were women (Table 1). The mean age was 47.5 years (±9.4), and the majority were aged 50 or older (49.6%). A total of 74.8% were married or cohabiting, and 76.5% had children. In terms of education, 23.5% were high school (Lyceum), Vocational Training Institute (IEK), or nursing school graduates; 46.2% were technical school graduates; 4.2% had a university degree; and 26.1% had a master’s degree. Mean years of work experience were 20.7 (±10.5).

### 3.2. Scores of the Quiet Quitting and Professional Quality of Life Scale Scores

Table 2 presents the Quiet Quitting Scale and Professional Quality of Life Scale scores for the 119 nursing staff participants in the present study. A high score (→5) indicates a higher level of quiet quitting. The mean total score was moderate-to-low mean (2.06 ± 0.59). Lack of Motivation (2.58 ± 0.97) scored significantly higher mean than Lack of Initiative (2.11 ± 0.67) or Distancing-Indifference (1.75 ± 0.62) (*p* < 0.001).

Regarding professional quality of life, Compassion Satisfaction (36.52 ± 6.29) scored moderate to high, with a higher mean than Burnout (27.60 ± 5.56), which was moderate, and Secondary Traumatic Stress (24.39 ± 6.73), which was moderate to low (*p* < 0.001).

**Figure 1:**
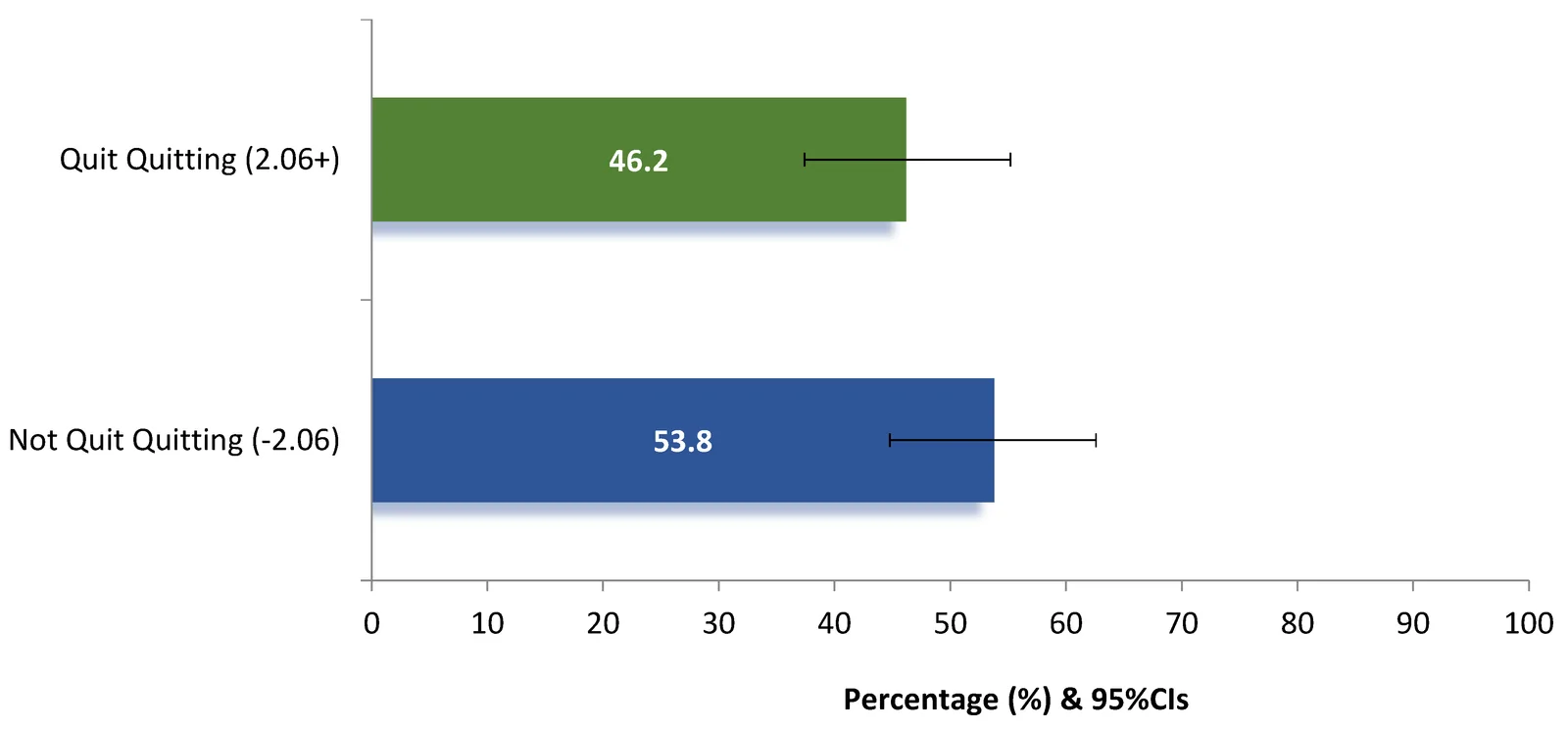
Increased incidence of quiet quitting from work among the 119 nurse participants in this study.

Fewer than half of the participants (46.2%, *p* > 0.05) presented increased quiet quitting (score > 2.06; Figure 1).

**Figure 2:**
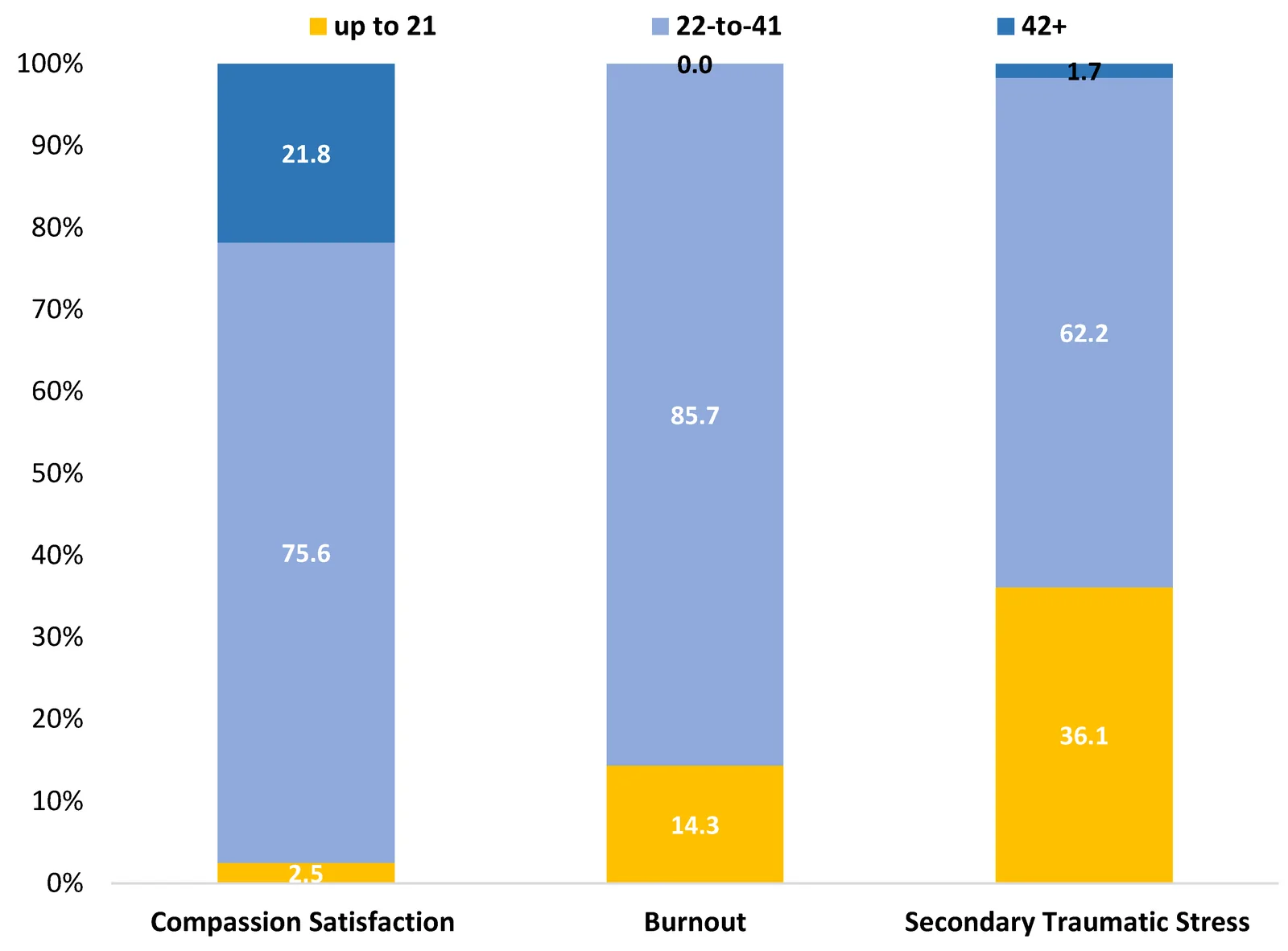
Frequencies of the graded distribution of the Professional Quality of Life subscales among the 119 participating nurses in the present study (higher scores indicate greater compassion but also greater burnout and stress).

Regarding the frequency distribution of the ProQuol subscales (Figure 2), 21.8% of nurses had high compassion satisfaction, while only 2.5% had low compassion satisfaction. None (0.0%) had high burnout, and only 1.7% had high stress.

Table 3 presents univariate correlations between professional quality of life and quiet quitting scores. Higher compassion satisfaction is significantly associated with lower total quiet quitting (r = −0.594, *p* < 0.05) and with lower scores on the Distancing-Indifference (r = −0.399, *p* < 0.05), Lack of Initiative (r = −0.465, *p* < 0.05), and Lack of Motivation (r = −0.622, *p* < 0.05) subscales. Higher total quiet quitting is also significantly associated with higher Burnout (r = 0.476, *p* < 0.05) and Secondary Traumatic Stress (r = 0.223, *p* < 0.05).

Table 4 presents the multiple linear regression examining relationships between scores on the three subscales of the ProQoL scale and the characteristics of the 119 participating nursing staff, as well as the Quiet Quitting Scale/subscales. It is estimated that higher levels of compassion satisfaction are significantly associated with lower levels of total quiet quitting (F = 11.80, df = 6, 12, unstandardized β = −6.23, *p* < 0.001) and are unrelated to staff characteristics (*p* > 0.05). Conversely, higher levels of burnout are significantly associated with higher levels of total quiet quitting (F = 6.07, df = 6, 12, β = 4.54, *p* < 0.001) but are unrelated to staff characteristics (*p* > 0.05). Finally, higher levels of secondary traumatic stress are significantly associated with female gender (F = 3.97, df = 6, 12, β = 4.92, *p* = 0.004) and with higher levels of total quiet quitting (F = 3.97, df = 6, 12, β = 3.40, *p* = 0.001).

## 4. Discussion

The primary aim of the present study was to assess the levels of quiet quitting and professional quality of life among nurses working in a public general hospital in Greece. The secondary aims were to (a) examine the associations between quiet quitting and professional quality of life; (b) assess whether work-related characteristics, such as workload, employment status, work experience, and working conditions, are associated with quiet quitting and professional quality of life; and (c) determine whether demographic characteristics, including age, gender, marital status, educational level, and other relevant sociodemographic factors, are associated with levels of quiet quitting and professional quality of life.

Overall, quiet quitting was found to be low to moderate. Among its three subscales, Lack of Motivation was notably higher than Lack of Initiative or Detachment-Indifference (*p* < 0.001). Similar results were reported in the original Greek validation cohort studies. Mean values for Detachment-Indifference, Lack of Initiative, and Lack of Motivation were 2.0, 2.3, and 2.8, respectively. Nurses experienced higher levels of lack of motivation and initiative and lower levels of detachment-indifference [21,25]. Another relevant cross-sectional study in Greece, surveying 186 healthcare professionals from a general hospital, revealed that 62% of participants exhibited characteristics of quiet quitting, with “Lack of Motivation” scoring highest [29]. Related results were reported in a study of 754 nurses across 13 Iranian hospitals, in which Lack of Motivation had the highest mean score, Lack of Initiative the second highest, and Detachment-Indifference the lowest among the Quiet Quitting subscales [30], as well as in a study on the scale’s adaptation and validation in the Portuguese nursing context [31]. A possible explanation is that motivation is the first factor in disengagement. Psychologically, it is easier to withdraw than to engage the behavioral component (initiative—going beyond one’s role) or the relational/affective component (detachment—actively disengaging from colleagues and the workplace). Employees may quietly reduce their inner drive long before it shows in behavior or interpersonal interactions. In the Iranian study mentioned earlier, the authors suggest that professional norms and role obligations (e.g., care duty, licensure accountability) serve as a partial buffer specifically against detachment, whereas motivational depletion is less externally policed and may therefore be reported at a comparatively higher level [30]. The increased incidence of lack of motivation among nurses with children, those with more years of experience, or those in a position of responsibility in the present study aligns with the findings of a relevant study [32], which notes that employee work engagement may be negatively affected by personal and professional obligations that accumulate over time.

Regarding the role played by the position of responsibility, two studies, one involving 186 healthcare professionals from a regional general hospital in Greece [29] and the other involving 425 Greek nurses recruited via social media [33], both linked quiet quitting to organizational and managerial variables in Greek hospital samples, suggesting that role-related exposure to conflict, workload, and insufficient organizational support, rather than years of experience or responsibility, may be an important factor.

The Job Demands–Resources (JD-R) model [34] explains these results, suggesting that employee well-being and engagement depend on the balance between job demands (such as workload, emotional labor, and role conflict) and job resources (such as autonomy, recognition, social support, and organizational backing). In this context, prolonged exposure to high job demands without sufficient resources may be associated with a harmful health process in which chronic stress is linked to gradually reduced motivation and depleted energy, while insufficient resources may be associated with weakened motivational systems that typically support engagement and initiative. The current findings align with the JD-R model’s health-impairment trajectory, in which unmet demands are associated with reduced motivation and reduced initiative or emotional disengagement.

This study supports that compassion satisfaction is associated with lower levels of burnout and quiet quitting, consistent with the model’s motivational trajectory. This pathway indicates that sufficient resources, including a sense of purpose, a feeling of achievement, and dedication to patient care, may be associated with lower disengagement and higher engagement even in challenging situations. The relationship between burnout and total quiet quitting highlights how inadequate resources may be linked to reduced energy, as seen in the health-deteriorating pathway. Moreover, heightened secondary traumatic stress in female nurses may reflect gender-specific inequalities in exposure to challenges, such as the requisite emotional labor, rather than merely a lack of resources. The JD-R model indicates that nursing staff facing increased demands and scarce resources may experience a gradual decrease in motivation. If left unaddressed, this may manifest as clear signs of disconnection, including diminished motivation or emotional aloofness, and may eventually lead to exhaustion or intentions to leave the field.

Among professional quality of life factors, Compassion Satisfaction scored a higher average (moderate to high) than Burnout (moderate) and Secondary Traumatic Stress (moderate to low) (*p* < 0.001). These findings align with a study examining the impact of moral resilience on quiet quitting, job burnout, and turnover intention among Greek nurses [21]. The study found compassion satisfaction to be a protective factor against quiet quitting and burnout. Researchers found that nurses who report high levels of compassion satisfaction are less likely to experience severe burnout symptoms, show greater work commitment, and have a lower intention to leave. The relevant literature [35,36] supports the view that quiet quitting serves as a strategy to reduce the psychological burden associated with constant exposure to human suffering. Similarly, employees who experience high levels of compassion fatigue often resort to distancing mechanisms, which can be associated with either resigning from work or quiet quitting from professional obligations [37]. Furthermore, a study examining job stress, burnout, and job satisfaction among staff working with people with intellectual disabilities [38] points out that burnout and secondary traumatic stress do not necessarily occur to the same extent, as secondary traumatic stress is influenced by factors such as the immediacy and nature of contact with traumatic incidents, which probably explains the findings of the present research.

These findings are consistent with a study on the Great Resignation in the post-COVID-19 era [39], which emphasizes that quiet quitting can be viewed as a mental self-protection mechanism against burnout. Related studies [26,30] also support this relationship, reporting that the emotional exhaustion accompanying burnout may be an important factor associated with employees’ withdrawal from active participation in their work environment. At the same time, the association of secondary traumatic stress with female gender, as shown in the present research, aligns with the findings of a study measuring compassion fatigue [37], which indicates that female health professionals are more emotionally burdened, which the study’s authors attributed to the increased empathy they develop toward patients. However, this finding should be interpreted with caution given the significant gender ratio disparity among nurses in the healthcare sector, where females outnumber males. Regarding the correlation between quiet quitting and professional quality of life among nurses, higher levels of compassion satisfaction were associated with lower levels of total quiet quitting. Higher levels of burnout were associated with higher levels of total quiet quitting, and higher levels of secondary traumatic stress were related to female gender and to higher levels of total quiet quitting. Convergent findings were reported in a study of 205 nurses at a Turkish university hospital examining relationships among stress of conscience, compassion fatigue, and quiet quitting [17]. The study found positive associations among all three, with compassion fatigue partially mediating the link between stress of conscience and quiet quitting. The current study’s finding that compassion satisfaction negatively predicts quiet quitting is the complementary, protective side of the same relationship.

Concerning the relationship between burnout and quiet quitting, a study of 1097 nurses in northern Portugal’s health services found a positive correlation between overall quiet quitting and burnout across all domains [40]. Regarding gender, a recent scoping review indicated that gender showed a significant positive correlation with secondary traumatic stress in some studies but not in others, highlighting its multifaceted and context-dependent nature [41]. This suggests that gender-related exposure to secondary traumatic stress in the current research outcomes may stem from the higher proportion of female nurses in the Greek clinical setting rather than from an inherent biological or dispositional vulnerability.

## 5. Limitations

This single-site cross-sectional study examined the effect of professional quality of life on quiet quitting among nurses in a general hospital. It also explored how work-related and demographic characteristics influence quiet quitting and professional quality of life. However, certain limitations should be acknowledged. The single-site cross-sectional design does not support causal inference about the relationships observed among quiet quitting, burnout, compassion satisfaction, and secondary traumatic stress. Longitudinal research is necessary to establish temporal precedence and to support the health-impairment and motivational pathways proposed within the JD-R framework. Regarding the sampling technique, the modest convenience sample from a single hospital limits the generalizability of the findings. Additionally, self-report data may introduce bias due to subjective responses. Furthermore, the sample’s gender imbalance, typical of the nursing workforce, limits gender-based comparisons, particularly regarding the association between female gender and secondary traumatic stress. The findings should be interpreted as exploratory rather than confirmatory. Despite their theoretical relevance in the JD-R model, potential confounding variables such as unit-level staffing adequacy, shift patterns, and perceived organizational support were not measured. Their omission may limit the explanatory completeness of the proposed pathways and warrants attention in future multicenter, prospective research.

## 6. Conclusions

Overall, quiet quitting was low to moderate. Among its three subscales, Lack of Motivation scored higher than Detachment-Indifference or Lack of Initiative. Additionally, nurses with children, those with more years of professional experience, or those in positions of responsibility presented higher incidences of lack of motivation. Regarding professional quality of life, Compassion Satisfaction had a higher average score (ranging from moderate to high) than Burnout, which was moderate, and Secondary Traumatic Stress, which ranged from moderate to low. Among nurses, higher compassion satisfaction correlated with lower total quiet quitting. Conversely, elevated levels of burnout were associated with increased levels of total quiet quitting, while higher levels of secondary traumatic stress were linked to female gender and higher levels of total quiet quitting.

These results highlight the need for interventions that primarily target motivational factors rather than burnout-related factors in quiet quitting, given that Lack of Motivation, rather than Detachment-Indifference, emerged as the predominant subscale. Educational interventions and competency-recognition programs may help sustain engagement among senior staff whose motivation may decline after years of routinized practice.

At the organizational level, given that elevated burnout was consistently associated with higher total quiet quitting in this sample, managers may benefit from communicating transparently and supporting employee motivation.

Future longitudinal studies may test the temporal and mechanistic pathways proposed by the Job Demands–Resources model. These studies should also include previously unmeasured but theoretically relevant confounders, such as unit-level staffing adequacy, shift-pattern intensity, and perceived organizational support.

## Figures and Tables

**Table 1:** Key characteristics of 119 nurses participating in this study.

		*n*	%
Gender	Males/Females	18/101	15.1/84.9
Age, years	Mean age ± SD	47.5 ± 9.4	
	50+	59	49.6
Marital Status	Single, Divorced, Widowed	30	25.2
	Married or cohabiting	89	74.8
Children	No/Yes	28/91	23.5/76.5
Education	Lyceum/IEK/Nursing School graduates	28	23.5
	Technical University	55	46.2
	University	5	4.2
	MSc	31	26.1
Work experience, years	Mean ± SD	20.7 ± 10.5	
Position of responsibility	No/Yes	105/14	88.2/11.8

**Table 2:** Mean scores of the Quiet Quitting subscales and Professional Quality of Life subscales among 119 nurses.

Scales & Subscales	Mean	SD	Median	Skewness	Cronbach’s α
Total Quiet Quitting (increased score ⇨ higher level of quiet quitting)	2.06	0.59	2.00	0.77	0.803
Detachment-Indifference	1.75	0.62	1.75	0.93	
Lack of Initiative	2.11	0.67	2.00	0.56	
Lack of Motivation	2.58	0.97	2.50	0.48	
Professional Quality of Life (increased score ⇨ a sign of compassion satisfaction but greater burnout & stress)					0.753
Compassion Satisfaction	36.52	6.29	37.00	−0.66	
Burnout	27.60	5.56	27.00	0.04	
Secondary Traumatic Stress	24.39	6.73	24.00	0.55	

Kruskal–Wallis Test of the three subscales on each Scale: *p* < 0.001.

**Table 3:** Correlation of the Quiet Quitting Scale and Professional Quality of Life Scale of the 119 participating nurses in the present study.

	Professional Quality of Life (Increased Score is a Sign of Compassion, but Greater Burnout and Stress)		
	Compassion Satisfaction	Burnout	Secondary Traumatic Stress
	r-Pearson (95%CIs)		
Total Quiet Quitting (increased score shows higher level of Quiet Quitting)	−0.594 * (−0.699, −0.464)	0.476 * (0.324, 0.604)	0.223 * (0.044, 0.387)
Detachment-Indifference	−0.399 * (−0.541, −0.236)	0.308 * (0.135, 0.462)	0.190 * (0.011, 0.358)
Lack of Initiative	−0.465 * (−0.595, −0.311)	0.322 * (0.151, 0.474)	0.215 * (0.037, 0.381)
Lack of Motivation	−0.622 * (−0.722, −0.498)	0.566 * (0.430, 0.677)	0.139 (−0.042, 0.312)

* *p* < 0.05.

**Table 4:** Multiple linear regression of the ratio of the scores of the three subscales of the ProQoL scale to the characteristics of the 119 participating nurses and total quiet quitting.

	Professional Quality of Life (Increased Score is a Sign of Compassion Satisfaction, but Greater Burnout and Stress)					
	Compassion Satisfaction		Burnout		Secondary Traumatic Stress	
Predictive Factors	β (Stand. Error)	*p*-Value	β (Stand. Error)	*p*-Value	β (Stand. Error)	*p*-Value
Gender (1: male, 2: female)	1.89 (1.33)	0.159	0.60 (1.30)	0.648	4.92 (1.65)	0.004
Children (1: no, 2: yes)	1.46 (1.21)	0.229	−1.29 (1.18)	0.279	−0.12 (1.50)	0.936
Education (1: Lyceum/IEK/Nursing School Graduates, 2: Technical University, 3: University, 4: MSc)	−0.44 (0.46)	0.340	0.41 (0.45)	0.364	−0.35 (0.57)	0.533
Work Experience (years)	−0.26 (0.24)	0.276	0.28 (0.24)	0.240	0.41 (0.30)	0.171
Position of responsibility (1: no, 2: yes)	0.43 (1.68)	0.800	−0.73 (1.65)	0.657	4.13 (2.09)	0.050
Total Quiet Quitting (higher score => higher level of quiet quitting)	−6.23 (0.82)	<0.001	4.54 (0.81)	<0.001	3.40 (1.02)	0.001
R^2^ (adjusted)	0.387 (0.354)		0.246 (0.205)		0.175 (0.131)	
F (d.f.)	11.80 (6, 112)		6.07 (6, 112)		3.97 (6, 112)	

β, unstandardized regression coefficient.

## Data Availability

The data presented in this study are available on request from the corresponding author. The data are not publicly available due to restrictions (privacy).
